# Age-dependent effects of carotid endarterectomy or stenting on cognitive performance

**DOI:** 10.1007/s00415-012-6491-9

**Published:** 2012-04-19

**Authors:** Katrin Wasser, Helmut Hildebrandt, Sonja Gröschel, Tomislav Stojanovic, Holger Schmidt, Klaus Gröschel, Sara M. Pilgram-Pastor, Michael Knauth, Andreas Kastrup

**Affiliations:** 1 Department of Neurology, University of Göttingen, Robert-Koch-Straße 40, 37075 Göttingen, Germany; 2 Department of Psychology, University of Oldenburg, Oldenburg, Germany; 3 Department of Thoracic Cardiovascular Surgery, University of Göttingen, Göttingen, Germany; 4 Department of Neuroradiology, University of Göttingen, Göttingen, Germany; 5 Departments of Neurology, Klinikum Bremen, Bremen, Germany; 6 Department of Neurology, University of Mainz, Mainz, Germany

**Keywords:** Carotid artery stent, Carotid artery endarterectomy, Cognition, Age

## Abstract

Although evidence is accumulating that age modifies the risk of carotid angioplasty and stenting (CAS) versus endarterectomy (CEA) for patients with significant carotid stenosis, the impact of age on cognition after either CEA or CAS remains unclear. In this study, we analyzed the effects of age on cognitive performance after either CEA or CAS using a comprehensive neuropsychological test battery with parallel test forms and a control group to exclude a learning effect. The neuropsychological outcomes after revascularization were determined in 19 CAS and 27 CEA patients with severe carotid stenosis. The patients were subdivided according to their median age (<68 years and ≥68 years); 27 healthy subjects served as a control group. In all patients clinical examinations, MRI scans and a neuropsychological test battery that assessed four major cognitive domains were performed immediately before, within 72 h, and 3 months after CEA or CAS. While patients <68 years of age showed no significant cognitive alteration after either CEA or CAS, a significant cognitive decline was observed in patients ≥68 years in both treatment groups (*p* = 0.001). Notably, this cognitive deterioration persisted in patients after CEA, whereas it was only transient in patients treated with CAS. These results demonstrate an age-dependent effect of CEA and CAS on cognitive functions. In contrast to the recently observed increased clinical complication rates in older subjects after CAS compared with CEA, CEA appears to be associated with a greater, persistent decline in cognitive performance than CAS in this subgroup of patients.

## Introduction

Carotid endarterectomy (CEA) is currently the accepted standard of treatment for patients with symptomatic and some selected patients with severe asymptomatic internal carotid artery stenosis [1, 2]. In recent years, however, carotid angioplasty and stenting (CAS) has emerged as an alternative endovascular treatment strategy for these disorders. While CAS has the main attractions of avoiding general anesthesia and surgical incisions reducing the incidence of wound problems or cranial nerve palsies, higher embolization rates during CAS compared to surgery have been reported using either transcranial Doppler sonography to monitor embolic events [3, 4] or diffusion-weighted imaging (DWI) to detect new embolic lesions after the intervention [5, 6]. In good agreement with these findings, several large randomized trials indicate that CAS is associated with a higher incidence of stroke at 30 days compared to CEA [7–11]. In contrast to the increased embolic complications rates after CAS compared to CEA, evidence is accumulating that both revascularization procedures lead to subtle cognitive impairment of similar magnitude.

In fact, we recently demonstrated that, although there is a higher burden of new ischemic brain lesions as detected with DWI after CAS, CAS was not associated with a greater, persistent cognitive decline compared to CEA [12]. Similar results were obtained in a subgroup study of the International Carotid Stenting Study (ICSS), which prospectively compared the effect of CEA or CAS on cognition in patients with symptomatic carotid artery stenosis [13]. Similar to our results, new ischemic lesions were found twice as often after CAS than after CEA in that study, but the cognitive changes between CAS and CEA were comparable [13]. Another small study has also corroborated these findings [14].

On the other hand, it is noteworthy that a measurable cognitive deterioration occurs in approximately 25 % of the patients irrespective of the treatment modality [15, 16]. Therefore, it is important to identify risk factors for these neurocognitive changes, all the more considering that many patients and especially those with an asymptomatic carotid stenosis, might only have a borderline indication for a revascularization. Previously, advanced age has been identified as a potential risk factor for neurocognitive decline after CEA [17], whereas the impact of age on cognition after CAS has not been specifically studied to date. Therefore, we analyzed the effects of age on cognitive performance after either CEA or CAS using a comprehensive neuropsychological test battery with parallel test forms and a control group to exclude a learning effect.

## Methods

### Patients

A total of 46 patients with high-grade carotid stenosis (≥70 % in symptomatic patients and ≥90 % in asymptomatic patients as assessed with ultrasound according to ECST criteria) were included in this analysis [18]. In all patients, the diagnosis of a high-grade carotid artery stenosis had been made by carotid duplex ultrasound using a combination of direct and indirect criteria and the presence and extent of intra- and post-stenotic turbulent flow. In detail, as direct criteria for the local degree of stenosis, the peak systolic flow velocities within the stenosis and post-stenotic internal carotid artery, the end diastolic flow velocity in the stenosis, the ICA/CCA ratio, and the pre- and post-stenotic frequency patterns were determined. The residual vessel lumen in the brightness mode image (B-image) and the color-coded residual vessel area were documented. As indirect criteria, the flow characteristics of the supratrochlear and anterior cerebral artery and the pulsatility of the common carotid artery were taken into account. As a key feature a local stenosis degree of ≥70 % was diagnosed if the peak systolic velocity exceeded 200 cm/s and a local stenosis degree of ≥90 % was diagnosed if the peak systolic velocity exceeded 400 cm/s. All examinations were performed in a standardized form in the same vascular laboratory with the same ultrasound equipment (Acuson Sequoia™ 512, Siemens, San José, CA) under the supervision of an experienced, board certified vascular neurologist (K.G.).

We have recently published a study that investigated the overall effects of new DWI lesions after either CEA or CAS on intellectual functions [12]. Now we performed a subgroup analysis of this dataset in order to evaluate the potential effect of age on cognitive functions after either CEA or CAS. The patients were subdivided according to the median age of the study population into two groups (<68 and ≥68 years). To avoid a negative influence on the test results, exclusion criteria were an arm palsy of the dominant side, hemianopsia, any type of expressive and/or receptive aphasia [patients exceeding 1 point of the item 9 (Best Language) of the National Institute of Health Stroke Scale (NIHSS)], poor German skills or a cognitive deficit of less than 26 points on the Mini-Mental State Examination (MMSE). All patients received detailed information about the potential risks and benefits of both CAS and CEA and were treated with either procedure based on their own individual decision. A carotid stenosis was considered symptomatic if the patient had experienced an ipsilateral ocular or cerebral (transient or permanent) ischemic event within the past 6 months. All patients gave their informed consent before participating in the study. The study had been approved by the Ethics Committee of the University of Göttingen, Germany.

### Control group

A total of 27 healthy subjects without a medical history of neurological or psychiatric disease, who were frequency matched for age (mean age ± SD: 65 ± 9 years) and length of school education, served as a neuropsychological control group. The test results of the control group were transformed into *z*-values, which served as reference for the patients.

### Carotid revascularization procedures

CAS was performed using a standardized protocol recently described in detail [19]. At least 3 days before the procedure, patients received orally administered aspirin (100 mg/day) and clopidogrel (75 mg/day). After stenting clopidogrel was continued for 6–12 weeks and aspirin was administered indefinitely. Cerebral angiography was restricted to the stent-preselected carotid artery and all stent procedures were performed by experienced senior neuroradiologists and done under anaesthesiological stand-by. According to physician preference and preinterventional evaluation of stenosis, 9/19 patients were treated with a filter-type protection device during CAS.

Experienced senior vascular surgeons performed all operations with the patient under general anesthesia. In 10/27 patients, intraoperative shunts had been used.

### Magnetic resonance imaging and analyses

In all patients, MRI scans were obtained immediately before, within 72 h, and 3 months after CEA or CAS. MRI was performed on a 3.0 Tesla MRI system (Siemens TIM Trio, Germany). Multi-slice diffusion-weighted single-shot EPI images and T2-weighted fluid attenuated inversion recovery turbo spin echo (FLAIR) images were acquired in all patients with parameters, which have recently been described in detail [12]. Either a CT angiography or a contrast enhanced MR angiography were performed prior to treatment in all subjects.

MR image analysis was performed jointly by a neuroradiologist (S.M. P.-P.) and a neurologist (K.G.) who were both blinded to the clinical data. All new DWI lesions were described by their number, location in the brain, and their size (given in mm^2^). The pre-interventional angiographies were used to decide if the new DWI lesions were inside or outside the vascular territory of the treated artery.

On the FLAIR images, the visual rating scale of Fazekas was used to determine the amount of periventricular and white matter hyperintensities (PVH and DWMH), respectively [20].

### Neuropsychological evaluations

The standardized neuropsychological test battery assessed four major cognitive domains, which are summarized in Table 1. The test battery was recently described in detail [12]. Briefly, attention functions were measured with two subtests of the “Testbatterie zur Aufmerksamkeitsprüfung” (TAP, “Tests for Attentional Performance”) [21, 22]. Verbal fluency tasks of the “Regensburger Wortflüssigkeitstest” (RWT, “Regensburger Word Fluency Test”) [23], the “Regard’s Five-Point Test” [24], and the Wisconsin Card Sorting Test (WCST) [25] were used to examine executive functions. Verbal learning and memory were tested with parts of the “Wechsler Gedächtnistest – revidierte Fassung” (WMS-R) [26] and of the Selective Reminding Test (SRT) [27]. Furthermore, non-verbal learning and memory were measured with the Rey–Osterrieth Complex Figure Test (ROCF) [28], “Non-Verbal Learning Test” (NVLT) [29], the “Spatial Recall Test” (SPART) [30], and the “Lern- und Gedächtnistest 3” (LGT-3) [31].

**Table 1:** Neuropsychological tests and cognitive domains

Attention
TAP subtest alertness
TAP subtest divided attention
Executive functions
WCST
5-Points test
RWT lexical fluency with and without alterations
Verbal learning/memory
Last trials and delayed recall of SRT
WMS-R logical memory
WMS-R verbal pair association
Non-verbal learning/memory
Delayed recall of Rey–Osterrieth Figure
SPART
LGT-3

Patients were examined at three time-points: in the hospital 1 day before (time-point 1; T1), and 1–4 days (time-point 2, T2), as well as 3 months (time-point 3, T3) after either CEA or CAS. To attenuate significant practice effects due to serial testing, we created a parallel version of our neuropsychological test battery using alternate forms available for most tests at follow-up immediately after revascularization. The same protocol was used in the control group. All subjects were tested individually and the tests were administered in the same order. All tests were either performed by a neurologist (K.W.) or a research assistant. Both were trained to administer and score the neuropsychological tests under the supervision of a physician experienced in neuropsychology (H.S.). They were blinded to the clinical outcome data, whereas they were not blinded as to the procedure performed.

Each test score was scaled to the normative data derived from the control group by *z*-transformation of the raw data. We calculated the *z*-scores for each cognitive domain by averaging the *z*-scores of its subtests and then averaged all *z*-scores of the neuropsychological tests to a compound for the cognitive status of each patient.

### Data collection and clinical evaluation

The following cerebrovascular risk factors were recorded using history or direct measurements: diabetes mellitus (HbA1c >6.5 %, fasting blood glucose >120 mg/dl or presence of antidiabetic drugs), arterial hypertension (blood pressure ≥140/90 mmHg measured on repeated occasions or presence of antihypertensive drugs), hyperlipidemia (fasting serum cholesterol levels >200 mg/dl or statin therapy), previous myocardial infarction, atrial fibrillation, previous transient ischemic attacks and strokes, and the presence of contralateral carotid stenosis ≥70 % or contralateral carotid occlusion (assessed with ultrasound or CT angiography).

Neurological examinations, the NIHSS and the Modified Rankin Score (mRS) were carried out in each patient by a stroke neurologist (A.K.) prior to CAS or CEA, the day after each procedure, and after 3 months. The definitions of post-interventional neurological complication rates that occurred within 30 days were defined as follows:

*Minor stroke* Any new neurological deficit (either ocular or cerebral) that persisted for more than 24 h and that either resolved completely within 30 days or increased the NIH stroke scale ≤3 points.

*Major stroke* Any new neurological deficit that persisted after 30 days or increased the NIH stroke scale by >3 points.

### Statistical analysis

Continuous values were expressed as mean ± SD and nominal variables as count and percentages. Median values and the interquartile range were computed as appropriate. For comparisons of categorical data two-tailed Chi-square statistics with Yates correction and univariate Fisher’s exact test were used. The Fisher’s exact test was used when the predicted contingency table cell values were less than five.

The averaged compound *z*-scores were analyzed by comparing the three time points, i.e., T1 (before CAS or CEA), T2 (1–3 days after CAS or CEA), and T3 (3 months after CAS or CEA) using repeated measures analyses of variance and the Greenhouse–Geisser correction.

The sequential assessments of the patients (time) was used as within subject factor, CAS or CEA (procedure), and aged below versus equal and above 68 years (median age) as between subjects factors, respectively.

A value of *p* < 0.05 was considered statistically significant. All statistical analyses were performed using, (Version 18, SPSS Inc).

## Results

A total of 19 patients were treated with CAS and 27 patients were treated with CEA. According to the procedure and their age, the population was divided into four subgroups (CAS patients <68 years: *n* = 12; CEA patients <68 years: *n* = 12; CAS patients ≥68 years: *n* = 7, and CEA patients ≥68 years: *n* = 15). The demographic and clinical characteristics of the four subgroups according to the procedure and their age are summarized in Table 2.

**Table 2:** Baseline characteristics of patients according to age and procedure

	Age <68 years CAS *n* = 12	Age <68 years CEA *n* = 12	Age ≥68 years CAS *n* = 7	Age ≥68 years CEA *n* = 15
Male sex	9 (75 %)	10 (83 %)	5 (71 %)	11 (73 %)
Median MMSE (IQR)	28.5 (28–30)	28 (26–29)	28 (28–29)	27.5 (26–28)
Cerebrovascular risk factors				
Diabetes mellitus	1 (8 %)	6 (50 %)	3 (43 %)	5 (33 %)
Arterial hypertension	11 (92 %)	10 (83 %)	6 (86 %)	13 (87 %)
Hyperlipidemia	10 (83 %)	11 (92 %)	5 (71 %)	10 (67 %)
Previous MI	2 (17 %)	2 (17 %)	1 (14 %)	5 (33 %)
Atrial fibrillation°	2 (17 %)	1 (8 %)	0 (0 %)	7 (47 %)
Presenting event				
Symptomatic stenosis	8 (67 %)	6 (50 %)	6 (86 %)	5 (33 %)
TIA*	6 (50 %)	3 (25 %)	4 (57 %)	2 (13 %)
Minor stroke	1 (8 %)	3 (25 %)	2 (29 %)	2 (13 %)
Major stroke	1 (8 %)	0 (0 %)	0 (0 %)	1 (7 %)
Median NIHSS (IQR)	0 (0–1)	0 (0–2)	0 (0–1)	0 (0–1)
Median mRS (IQR)	0 (0–1)	0 (0–1)	0 (0–0)	0 (0–0)
Lesion characteristics				
Contralateral ICA stenosis ≥70 %	1 (8 %)	4 (33 %)	1 (14 %)	1 (7 %)
Contralateral ICA occlusion	1 (8 %)	0 (0 %)	0 (0 %)	1 (7 %)

* Significant difference between all four groups after post hoc analysis

° Significant difference between the two groups of patients ≥68 years after post hoc analysis

With respect to the baseline characteristics, the four groups did not differ for sex, MMSE, diabetes mellitus, arterial hypertension, hyperlipidemia, previous myocardial infarction, symptomatic stenosis, previous strokes, NIHSS, mRS, and contralateral stenosis or occlusion. However, we detected significant differences between all four groups with respect to previous TIA (*p* = 0.011) and atrial fibrillation (*p* = 0.031). Post hoc *T* tests for independent measurements showed a significant difference between the younger patient groups with respect to previous TIA (*p* = 0.013) and between the older patient groups with respect to previous TIA (*p* = 0.032) and atrial fibrillation (*p* = 0.029).

In this series, one patient (44 years), who was treated with CAS, developed a TIA post-interventionally. There were no further minor or major strokes after either CEA or CAS.

Six patients (<68 years: 3; ≥68 years: 3) did not have an MRI scan within 48 h after treatment (either declined or due to scheduling difficulties) and five patients (<68 years: 1; ≥68 years: 4) did not have a 3-month follow-up MRI.

Before each procedure, diffusion-weighted imaging revealed ischemic lesions in 5/16 (31.2 %) of the patients treated with CAS and in 5/26 (19 %) of the patients treated with CEA (*p* = 0.5). While just one CEA patient (1/24, 4.2 %) had a new DWI lesion postoperatively, new DWI lesions were detected among 11/16 (67 %) of the CAS patients immediately after treatment (*p* < 0.001). The incidence of new DWI lesions after CAS was significantly higher in patients ≥68 years of age (6/6; 100 %) than in younger patients (5/10; 50 %, *p* = 0.04). Post hoc *T* tests for independent measurements revealed a significant difference with regard to the occurrence of new DWI lesions as well between the two groups <68 years (*p* = 0.007) as between the two groups ≥68 years (*p* < 0.001). The scores according to Fazekas [20] did not show a significant difference between the four groups. The MRI findings are summarized in Table 3.

**Table 3:** MRI characteristics of patients according to age and procedure

	Age <68 years CAS	Age <68 years CEA	Age ≥68 years CAS	Age ≥68 years CEA
New DWI lesions*	*n* = 10	*n* = 11	*n* = 6	*n* = 13
	5 (50 %)	0 (0 %)	6 (100 %)	1 (8 %)

Fazekas score	*n* = 11	*n* = 12	*n* = 7	*n* = 13
Periventricular hyperintensity				
Grade 0	5 (45 %)	4 (33 %)	1 (14 %)	1 (8 %)
Grade 1	2 (18 %)	4 (33 %)	1 (14 %)	6 (46 %)
Grade 2	2 (18 %)	3 (25 %)	4 (57 %)	3 (23 %)
Grade 3	2 (18 %)	1 (8 %)	1 (14 %)	3 (23 %)
Deep white matter hyperintense signals				
Grade 0	3 (27 %)	4 (33 %)	3 (43 %)	4 (31 %)
Grade 1	2 (18 %)	4 (33 %)	2 (29 %)	4 (31 %)
Grade 2	4 (36 %)	3 (25 %)	4 (57 %)	3 (23 %)
Grade 3	2 (18 %)	1 (8 %)	2 (29 %)	1 (8 %)

* Significant difference between all four groups after post hoc analysis

Repeated measures analyses of variance revealed significant main effects for (time) [*F*(2,41): 11,712; *p* < 0.01] and for (median age) [*F*(1,42); *p* < 0.001], but not for (procedure). The twofold interaction of (time) × (procedure) [*F*(2,41): 5,392; *p* = 0.006], and the threefold interaction of (time) × (procedure) × (median age) [*F*(2,41): 8,535; *p* = 0.001] were also significant.

**Fig. 1:**
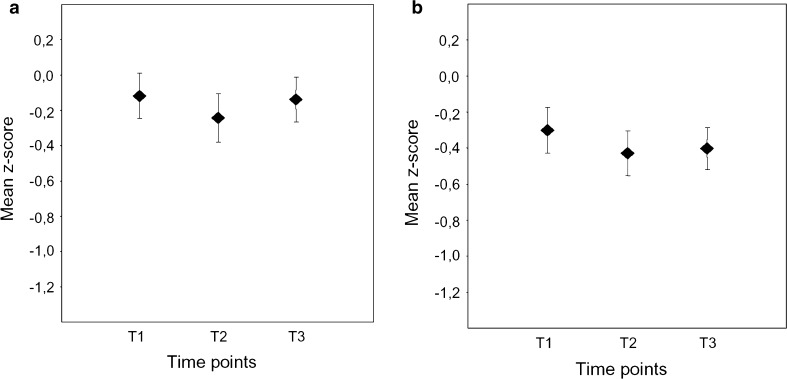
Mean compound *z*-scores (±SEM) in younger patients (<68 years) after carotid endarterectomy (**a**) or stenting (**b**) prior to treatment (T1), 1–3 days after treatment (T2), and 3 months after treatment (T3)

**Fig. 2:**
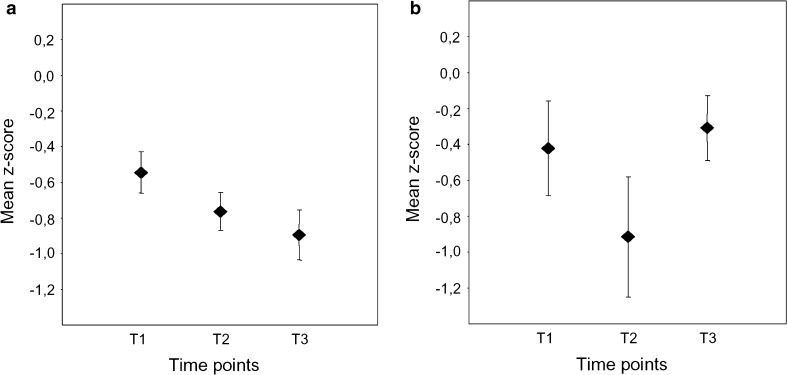
Mean compound *z*-scores (±SEM) in older patients (≥68 years) after carotid endarterectomy (**a**) or stenting (**b**) prior to treatment (T1), 1–3 days after treatment (T2), and 3 months after treatment (T3)

The mean changes of the *z*-values of the cognitive compound score at each of the three time points are summarized in Figs. 1 and 2. While patients <68 years of age showed no significant cognitive alteration after either CEA or CAS, a significant cognitive decline was observed in patients ≥68 years. Notably, this cognitive deterioration persisted in patients after CEA, whereas it was only transient in patients treated with CAS.

With respect to the changes of the cognitive compound score and at all three time-points post hoc *T* tests for independent measurements revealed no significant differences between the younger CEA and CAS patients. While the older CAS and CEA patients had comparable cognitive compound scores immediately before and after treatment, the older CEA patients were cognitively more impaired after 3 months than the older CAS patients (*p* < 0.05). This difference was larger than half of a standard deviation and the CEA group differed from the healthy controls of about −0.9 SD.

Intragroup dependent *T* tests for the three assessments demonstrated that the older group treated with CAS significantly deteriorated between T1 and T2 (*p* = 0.01), but also improved in cognitive performance between T2 and T3 (*p* = 0.017). Therefore, T1 and T3 did not differ for this group. Older patients treated with CEA showed a significant decline between T1 and T2 (*p* = 0.022) and also between T1 and T3 (*p* = 0.002). Similar results were obtained using non-parametric Mann–Whitney *U* Tests and Wilcoxon Tests.

## Discussion

In this study, we analyzed the impact of age on cognition after either CEA or CAS using a comprehensive neuropsychological test battery with parallel test forms and a control group to exclude a learning effect. Our results demonstrate an age-dependent effect of CEA and CAS on cognitive functions. While patients <68 years of age showed no significant cognitive alteration after either CEA or CAS, a significant cognitive decline was observed in patients ≥68 years. This decline in cognitive function was transient after CAS, whereas it persisted in patients after CEA. In contrast to the recently observed increased clinical complication rates in older subjects after CAS compared with CEA [7, 32, 33], CEA appears to be associated with a greater decline in cognitive performance than CAS in this subgroup of patients.

To date, several studies have tried to clarify the impact of carotid revascularization on cognition, but contradictory results have been found [34]. At least partly, these discrepant results are caused by methodological differences among the various studies including patient selection, presence of a control group, number and type of cognitive tests, and statistic measures among others. Despite these limitations, discrete declines in cognitive functions immediately after CEA and during long-term follow-up have been reported repeatedly [15, 35–39]. This finding principally is in good agreement with our results.

We could show that older patients suffer from a significant cognitive decline after either CEA or CAS. Notably, this decline in cognitive function in older patients was transient after CAS, whereas it persisted in patients after CEA. In good agreement with the latter finding, advanced age was a significant predictor for persistent neurocognitive dysfunction in a previous study, which had enrolled 186 CEA patients [17]. Advanced age is also a well-known predictor of cognitive decline after cardiac surgery [40]. We are not aware of any published studies which have specifically analyzed the impact of age on cognitive outcome after CAS. Irrespective of age, Gaudet et al. recently also reported a transient decline in cognitive performance early after CAS with a measurable improvement after 1 month [41]. It remains unclear, why cognitive functions initially declined early after treatment and then subsequently improved during follow-up in older CAS patients, whereas they also declined early after treatment and then deteriorated further in older CEA patients during follow-up. While many researchers favor the hypothesis that microemboli are the cause of neuropsychological signs after carotid revascularization, we and others recently showed that new brain lesions as detected with DWI after CAS or CEA do not affect long-term cognitive performance [12, 13, 42]. Similarly, Heyer et al. [38] also failed to show an association between cognitive decline and DWI lesions after CEA. On the other hand, we did observe transient cognitive decline in patients with new DWI lesions early after carotid revascularization irrespective of age in our previous study [12]. In this study, the incidence of new DWI lesions after CAS was also significantly higher in older than in younger patients (6/6, 100 % vs. 5/10, 50 %; *p* = 0.04) and advanced age has been shown to be a major risk factor for new DWI lesions after CAS [43, 44]. Therefore, it could be speculated that the initial decline in cognitive performance in older patients after CAS is at least partly attributable to cerebral microembolism. The improvement of cognitive performance during follow-up could then reflect the common observation that the vast majority of new DWI lesions after CAS are small and do not cause permanent ischemic damage [5, 12, 45, 46].

Aside from the dislodgement of microemboli, the observed decline in cognitive functions in older CEA patients could also be due to transient hypoperfusion during carotid cross-clamping or even longer lasting blood flow abnormalities after CEA. A close relationship between a hemodynamic dysregulation and post-CEA cognitive dysfunction was recently reported [47]. Older CEA patients could, thus, be particularly vulnerable to the hemodynamic alterations during the time of carotid artery cross-clamping, as well as in the early postoperative period.

The use of general anesthesia could also have contributed to the cognitive decline in the group of CEA patients. However, the results of studies investigating the effect of local or general anesthesia on cognitive functions after CEA are contradictory. In a subgroup analysis of the GALA study, the postoperative neurocognitive performance in the Trail Making Test decreased significantly in the general anesthesia group, whereas there were no significant changes in the local anesthesia group [48]. Furthermore, significantly higher levels of S100β as a marker of blood–brain barrier function and brain lesions were detected in the general anesthesia group compared to the local anesthesia group in that study [47]. In contrast to these findings, the incidence of cognitive deterioration after CEA did not differ between two groups of patients undergoing CEA with general or regional anesthesia a recent study by Heyer et al. [48]. Although a potential age-related interaction between general anesthesia and cognitive outcome after CEA has not been specifically studied, the use of general anesthesia could at least partially have contributed to the cognitive decline in the older CEA patients. In support of this notion, advanced age was a risk factor for cognitive dysfunction 3 months after major noncardiac surgery in the International Study of Post-Operative Cognitive Dysfunction study [49].

Finally, it is noteworthy that the incidence of atrial fibrillation was significantly higher in the group of older CEA patients than in the group of older CAS patients. Since the presence of atrial fibrillation has been shown to be associated with neurocognitive dysfunction after coronary artery bypass grafting [50], it could be speculated that this factor also contributed to the observed cognitive deterioration among the older CEA patients.

Strengths of our study include the evaluation of a control group at all three time points, as well as the use of parallel versions for the majority of the cognitive tests. On the other hand, we acknowledge that our study has inherent limitations imposed by its retrospective analysis, the relatively small sample size, and the non-randomization of treatment allocation. Furthermore, it could be questioned whether the observed cognitive decline in the older CEA patients is functionally relevant. Yet, in previous studies a decline of 0.5 standard deviations, as observed in this study between the older CEA and CAS patients, has also been considered as clinically relevant loss in cognitive function [51, 52]. Comijs et al. [53] showed that a cognitive decline of 0.5 standard deviation in the Mini-Mental Status Examination reflects about 6 years of aging in a representative group of older, healthy subjects. Finally, it should be pointed out that a standardized neuromonitoring had not been performed in the CEA patients, who had all been treated with general anesthesia.

Despite these limitations, our study has important clinical implications. Our results demonstrate an age-dependent effect of CEA and CAS on cognitive functions. In contrast to the recently observed increased clinical complication rates in older subjects after CAS compared with CEA, CEA appears to be associated with a greater, persistent decline in cognitive performance than CAS in this subgroup of patients. If confirmed in larger data sets, these results should be considered in weighing the risks and benefits of CEA, especially in older patients with an asymptomatic carotid stenosis.
